# Monthly Summary

**Published:** 1893-01

**Authors:** 


### Monthly Summary.

PRODUCTION OF PLATINA IN RUSSIA.—The platina veins in the Ural mountains are unique in that this metal is found there in grains. Platina is also found in Brazil and in the Cordilleras, but in none of these places is it obtained in grains. In Goroblagodatsky there are sixty-six mineralogical societies for the exploitation of platina, which search the coasts of Touri river and its tributaries.

The demand for platina, which increases every day, dates only from twelve to thirteen years ago. During the past decade the output of the metal has diminished till about 3,194 kilo. annually, more than half being obtained from the northern veins of the Ural mountains, which belong to the Empire. The total consumption of the world reaches 3,270 kilo., but the increase of the manifold applications of this metal in industries must increase that figure considerably.

When the demand for platina is insignificant and the price very low, the miners who find it when searching for gold use it for small shot on hen-birds. All the platina which is obtained from the Ural mountains, after the payment of a tax of three per cent. in material, is sent in a rough state to St. Petersburg, to be shipped to foreign markets.—*La Odontologia.*

---

VARIOUS BLOW-PIPE FLAMES.—Thomas Fletcher, the well-known expert in practical metallurgy, gives the following directions for the use of the blow-pipe:

The flames may be separated into two classes. Those used for blow-pipe analysis are produced by air jets of small bore, and as smooth as possible inside; the theoretically perfect jet is made of glass tube drawn out small, and broken off where the required bore exists. The advantages of this jet is the perfect smoothness of the bore, which enables the operator to produce perfectly defined flames, with the reducing and oxidizing zones large and clearly defined. The disadvantage of the glass jet is its delicacy and constant liability to injury. Next to this comes the platinum tip, which remains fairly smooth inside; and last of all comes the simple and cheap brass nozzle so universally used. The flame in this class of blowpipe is produced by an air pressure low enough to prevent the breaking up of the blue cone, the tip of which is the hottest part; inside this blue cone is the reducing flame; beyond it is the oxidizing zone. For brazing and soldering, a heavier air pressure and a larger bore jet are required; the blue cone is broken up, and the different zones of flame are much less clearly defined, and in practice are much less important. The flame is still roughly divided into a blue, or greenish-bl e center, and an outer yellowish mantle, surrounding and projecting beyond the blue. The rough point of the latter is, as before, the hottest part, and this should touch the work to be brazed. It has a distinctly oxidizing action; but this is overcome in practice by the protection of the flux used, which must have the power of dissolving oxides.—*Dental Pract. and Advertiser.*

---

FAILURE IN CROWN WORK.—By H. B. Meade, D.D.S., Buffalo, N. Y.—Many articles have been written concerning the failures made in banding roots for crown work, and many dentists have abandoned the use of collars or bands entirely, because a few cases have come under their observation in which a band was poorly adjusted, there being a V-shaped space between the band and root, the parts being in a highly inflamed condition. If bands are to be fitted in this manner, it would be better to discontinue the use of them at once, but with the exercise of intelligent care, no such space is necessary.

We must bear in mind that we are dealing with one of the most delicate tissues of the human anatomy, when we extend a band so far up the root that it interferes with the peridental membrane. If we take into consideration the anatomical form of any of the anterior roots, after the crown has been excised and ground down to the gum line, and then imagine the fitting of a band to nothing more or less than a cone, commencing at its base, we will at once see how the V-

shaped space is produced, as the band must necessarily be as large as the end of root, that it may be driven on.

To overcome the V-shaped space, and have the parts remain in a healthy condition, the band must not be driven on a root that has had no other preparation than merely the crown excised and ground down to the gum-margin. The remaining portion of the enamal that lies under the free margin of the gum must be removed, care being taken so to shape the root that its sides will be parallel, and the band must not extend further than the free margin of the gum. A band adjusted in this manner (using pure gold) will cause no inflammation. The end of the roof should be ground concave from labial to palatal surfaces, giving it the same curvature as the gum line. This will make the fitting of the porcelain crown to the band an easy matter.—*Dental Pract. and Advertiser.*

---

DRYING OF PAINTS.—We commonly speak of paints as "drying," and the term is entirely correct from one point of view. Yet it is not a process of dehydration, for, of course, there is no water to evaporate. It is a chemical process, the oxygen of the air combining with the oil to make a solid body. In the course of this oxidation the coloring matter of the paint mixture is fixed in the solidified oil, and hence the tint is permanent as long as the oil lasts. The preservative qualities of paint are due to the fact that the oil and pigmentary matter forms an impenetrable coating.—*Dental Pract. and Advertiser.*

---

WHEN WE SEE a young dentist peering round here and there, sparing no pains to find "the lay of the land" in every direction, and insinuating himself into the good graces of every one he meets, and becoming a favorite with old dentists, concealing nothing and learning of all, we make up our mind this young man is going to succeed. But when we see one coming home from college in a dandy suit, to be admired and waited on, and sitting down in the parlor in egotism and self-consciousness, to wait for rich patients, we do not count on him.—*Items of Interest.*

